# Beyond the typical immune effector cell-associated neurotoxicity syndrome: Characterizing non-immune effector cell-associated neurotoxicity syndrome neurological complications in chimeric antigen receptor T-cell therapy—A retrospective analysis

**DOI:** 10.1093/noajnl/vdag204

**Published:** 2026-08-05

**Authors:** Melissa A Nicholas, Abdulaziz Abu-Haimed, Faisal Abushullaih, Emily N Davidson, Graeme A Fenton, Justin W Kehm, Hakan Kocoglu, Jean A Yared, Ariel Fromowitz, Nancy M Hardy, Aaron P Rapoport, Djordje Atanackovic, Lily Pham, Ashraf Badros, Haroon Ahmad

**Affiliations:** Department of Medicine, University of Maryland School of Medicine, Baltimore, Maryland, USA; Department of Medicine, University of Maryland School of Medicine, Baltimore, Maryland, USA; Department of Medicine, University of Maryland School of Medicine, Baltimore, Maryland, USA; Department of Medicine, University of Maryland School of Medicine, Baltimore, Maryland, USA; Department of Medicine, University of Maryland School of Medicine, Baltimore, Maryland, USA; Department of Medicine, University of Maryland School of Medicine, Baltimore, Maryland, USA; Department of Medicine, University of Maryland School of Medicine, Baltimore, Maryland, USA; Department of Medicine, University of Maryland, Greenebaum Comprehensive Cancer Center, Baltimore, Maryland, USA (H.K., J.A.Y., A.F., N.M.H., A.P.R., D.A., L.P., A.B., H.A.); Transplant and Cellular Therapy Program, University of Maryland Greenebaum Comprehensive Cancer Center, Baltimore, Maryland, USA; Department of Medicine, University of Maryland School of Medicine, Baltimore, Maryland, USA; Department of Medicine, University of Maryland, Greenebaum Comprehensive Cancer Center, Baltimore, Maryland, USA (H.K., J.A.Y., A.F., N.M.H., A.P.R., D.A., L.P., A.B., H.A.); Transplant and Cellular Therapy Program, University of Maryland Greenebaum Comprehensive Cancer Center, Baltimore, Maryland, USA; Department of Medicine, University of Maryland School of Medicine, Baltimore, Maryland, USA; Department of Medicine, University of Maryland, Greenebaum Comprehensive Cancer Center, Baltimore, Maryland, USA (H.K., J.A.Y., A.F., N.M.H., A.P.R., D.A., L.P., A.B., H.A.); Transplant and Cellular Therapy Program, University of Maryland Greenebaum Comprehensive Cancer Center, Baltimore, Maryland, USA; Department of Medicine, University of Maryland School of Medicine, Baltimore, Maryland, USA; Department of Medicine, University of Maryland, Greenebaum Comprehensive Cancer Center, Baltimore, Maryland, USA (H.K., J.A.Y., A.F., N.M.H., A.P.R., D.A., L.P., A.B., H.A.); Transplant and Cellular Therapy Program, University of Maryland Greenebaum Comprehensive Cancer Center, Baltimore, Maryland, USA; Department of Medicine, University of Maryland School of Medicine, Baltimore, Maryland, USA; Department of Medicine, University of Maryland, Greenebaum Comprehensive Cancer Center, Baltimore, Maryland, USA (H.K., J.A.Y., A.F., N.M.H., A.P.R., D.A., L.P., A.B., H.A.); Transplant and Cellular Therapy Program, University of Maryland Greenebaum Comprehensive Cancer Center, Baltimore, Maryland, USA; Department of Medicine, University of Maryland School of Medicine, Baltimore, Maryland, USA; Department of Medicine, University of Maryland, Greenebaum Comprehensive Cancer Center, Baltimore, Maryland, USA (H.K., J.A.Y., A.F., N.M.H., A.P.R., D.A., L.P., A.B., H.A.); Transplant and Cellular Therapy Program, University of Maryland Greenebaum Comprehensive Cancer Center, Baltimore, Maryland, USA; Department of Medicine, University of Maryland, Greenebaum Comprehensive Cancer Center, Baltimore, Maryland, USA (H.K., J.A.Y., A.F., N.M.H., A.P.R., D.A., L.P., A.B., H.A.); Department of Neurology, University of Maryland School of Medicine, Baltimore, Maryland, USA; Department of Medicine, University of Maryland School of Medicine, Baltimore, Maryland, USA; Department of Medicine, University of Maryland, Greenebaum Comprehensive Cancer Center, Baltimore, Maryland, USA (H.K., J.A.Y., A.F., N.M.H., A.P.R., D.A., L.P., A.B., H.A.); Transplant and Cellular Therapy Program, University of Maryland Greenebaum Comprehensive Cancer Center, Baltimore, Maryland, USA; Department of Medicine, University of Maryland, Greenebaum Comprehensive Cancer Center, Baltimore, Maryland, USA (H.K., J.A.Y., A.F., N.M.H., A.P.R., D.A., L.P., A.B., H.A.); Department of Neurology, University of Maryland School of Medicine, Baltimore, Maryland, USA

**Keywords:** CAR-T therapy, cytokine release syndrome, ICANS, immunotherapy, neurotoxicity

## Abstract

**Background:**

Chimeric antigen receptor (CAR) T-cell therapy has revolutionized the treatment of relapsed/refractory hematologic malignancies. While immune effector cell-associated neurotoxicity syndrome (ICANS) is a well-recognized complication, expanding clinical use has revealed distinct non-ICANS neurological complications that challenge standard diagnostic and management approaches. We characterize the clinical features and outcomes of these non-classic presentations.

**Methods:**

A retrospective analysis was conducted on 357 patients receiving commercial CAR-T therapy for non-Hodgkin lymphoma or multiple myeloma at a single academic center (2015-2025). Non-ICANS neurological complications were defined by neurologic deficits, movement disorders, or neurocognitive syndromes occurring in the absence of global encephalopathy (ICE scores 9-10). Institutional protocol included baseline neurology evaluation and brain MRI.

**Results:**

Non-ICANS complications occurred in 17 patients (4.76%). Manifestations included focal neuropathies or myelopathy (*n* = 5), progressive leukoencephalopathy (*n* = 4), neuropsychiatric symptoms (*n* = 3), Parkinsonism (*n* = 2), and cranial nerve palsies (*n* = 4). Compared to the institutional median for classic ICANS (7 days), non-ICANS demonstrated a prolonged median duration of 14 days (range, 4-365+ days). Abnormal brain MRI findings were present in 5 of 15 (33.3%) cases with post-treatment brain MRI, manifesting commonly as T2/FLAIR hyperintensities in the white matter. Cerebrospinal fluid (CSF) immunomonitoring in a subset of cases (*n* = 2) revealed an enrichment of CAR T-cells compared to peripheral blood.

**Conclusions:**

Non-ICANS neurological complications represent a distinct clinical subset characterized by atypical findings, neuroimaging abnormalities, and/or protracted recovery. Increased clinical vigilance and tailored diagnostic approaches are essential to mitigate long-term morbidity, as standard screening tools fail to capture these focal neurotoxicities.

Key Points

- Non-ICANS affects ∼5% of CAR-T patients and can present in a variety of ways.
- Symptoms are associated with significantly prolonged recovery times.
- Non-ICANS syndromes can be a diagnostic dilemma if clinicians are unaware of these presentations.

Importance of the Study

While immune effector cell-associated neurotoxicity syndrome (ICANS) is well-characterized, its non-ICANS manifestations—ranging from isolated cranial neuropathies to progressive leukoencephalopathy—remain poorly defined and frequently result in diagnostic delays. This study characterizes a distinct cohort of patients with atypical neurotoxicity, demonstrating that these presentations are associated with significantly more protracted recovery times than classic ICANS. Furthermore, we provide evidence using cerebrospinal fluid (CSF) immunomonitoring, suggesting a methodology for future translational studies in chimeric antigen receptor (CAR)-T neurotoxicity. These findings expand the current neurotoxicity paradigm and provide clinicians with a framework for early recognition and management of non-stereotypical ICANS. By identifying high-risk phenotypes and potential biomarkers in the CSF, this work informs more personalized monitoring and intervention strategies to mitigate long-term neurological morbidity in cellular immunotherapy.

Non-Hodgkin lymphoma (NHL) and multiple myeloma (MM) are hematologic malignancies with average 5-year survival rates of 74% and 64%, respectively.^1^^,^^2^ Both are increasing in prevalence due to increased incidence, older patient populations, and longer overall survival (OS) secondary to advances in treatment.^3^^,^^4^ Initial treatment regimens include a combination of chemotherapy and monoclonal antibodies followed by stem cell transplantation. In patients with relapsed or refractory disease, chimeric antigen receptor T-cell therapy (CAR-T) is an emerging option. This personalized immunotherapy involves genetically engineering a patient’s T-cells to target cancer cell antigens, such as CD19 in B-cell lymphomas and B-cell maturation antigen (BCMA) in MM. The cells are proliferated in-vitro and reinfused in a controlled setting. CAR-T therapy has led to improved response rates and OS in patients who had exhausted traditional treatment options.^5^^,^^6^ Several CAR T-cell therapies have received regulatory approval, and many other engineered cell therapy products are in development for both hematologic and solid tumor malignancies. CAR-T therapy is a key player in the paradigm shift toward immuno-oncologic treatments.

Despite its efficacy, CAR-T therapy is associated with well-documented toxicities: cytokine release syndrome (CRS) and immune effector cell-associated neurotoxicity syndrome (ICANS). CRS occurs in roughly 75% of patients and presents with fever, hypotension, and/or hypoxia.^7^ ICANS is a well-recognized neurological complication occurring in approximately 20%-70% of patients treated with CAR-T cell therapy.^8^ It typically manifests within days to weeks post-infusion, concurrently or following CRS. ICANS can range from mild neurologic symptoms, such as disorientation, language disturbances, and tremor, to more severe manifestations, including delirium, seizures, cerebral edema, obtundation, coma, and death.^9^ Management of ICANS is guided by severity and often includes a combination of supportive care, anti-seizure medications, and immunosuppressants. The mainstay of immunosuppression is corticosteroids, but in refractory cases interleukin-6 (IL-6) and IL-6 receptor antibodies have been used.^10^ While the symptoms can be severe, ICANS is generally reversible, with patients experiencing recovery in days to weeks with appropriate intervention.

The nature of classic ICANS presentation is well-established, as are standardized treatment protocols.^11^ However, the increased use and expanding application of CAR-T therapy has led to the recognition of less common, non-ICANS neurological complications. Documented cases increasingly align with emerging classifications such as those proposed by the European Society for Blood and Marrow Transplantation (EBMT), including movement and neurocognitive toxicities (MNT), tumor inflammation-associated neurotoxicity (TIAN), cranial nerve palsies, and myelopathies.^12-15^ These events extend beyond the stereotyped ICANS presentation, making timely diagnosis and intervention challenging. Furthermore, differentiating between on-target/on-tumor, off-tumor/on-target mechanisms, and secondary neuroinflammatory processes remains an ongoing area of investigation.

Such neurological sequelae underscore the need for systematic reporting. Comprehensive reporting will enhance the understanding of the spectrum of CAR-T-associated neurotoxicity and inform the development of more targeted management strategies. We report a single-center series of 17 patients with non-ICANS neurological complications, demonstrating that these presentations are often associated with prolonged symptom duration and distinct focal findings, highlighting the need for tailored diagnostic and management approaches.

## Methods

A retrospective chart review was conducted on patients with MM or NHL who received commercial CAR-T cell therapy at a single academic center between 2015 and 2025. Cases of non-ICANS neurological complications were reviewed to characterize clinical features, preexisting neurologic disease, timing, diagnostic workup, treatment, and outcomes. An Institutional Review Board (IRB) exemption was obtained. Descriptive statistical analysis was performed using Statistical Package for the Social Sciences (SPSS).

As part of institutional protocol, all patients receiving CAR-T therapy had a baseline neurologic assessment, including mental status exam. All patients also received a brain MRI unless they had specific contraindications to MRIs, in which case they had CT scan of the head was completed. All patients receiving CAR-T had CRS/ICANS grading recorded every 8 hours throughout the admission, so this data was collected in real-time, not assigned in retrospect. The institutional protocol also includes 24-hour continuous EEG monitoring for patients with suspected ICANS grade 2 or greater. So, in all our patients, non-convulsive status epilepticus (NCSE) and epileptic seizures were ruled out.

To ensure that the identified neurological complications were uniquely associated with CAR-T cell therapy, a comprehensive diagnostic workup was systematically reviewed for all included patients to exclude alternative clinical etiologies. Metabolic delirium, systemic infections, and medication-induced toxicities were ruled out via continuous monitoring of metabolic panels, systemic cultures, and medication reconciliation. To differentiate neurotoxicity from structural, vascular, or treatment-induced changes, baseline pre-CAR-T neuroimaging (brain MRI or CT) was strictly compared against post-treatment event-driven imaging. Furthermore, disease progression was systematically ruled out in all cases through longitudinal clinical assessment, stable neuroimaging, and cytological evaluation of the CSF where clinically indicated.

A case was classified as a non-ICANS neurological complication if the primary or predominant neurological manifestation fell outside the typical, previously characterized symptoms of global encephalopathy. Specifically, this included isolated focal neurologic deficits (eg, cranial nerve palsies), movement disorders, cognitive/psychiatric symptoms distinct from global encephalopathy, progressive neuroimaging syndromes, and specific myelopathies. Duration of the complication was defined as the number of hospital admission days that the patient had a CARTOX score less than 10 and/or documentation of persistent, atypical neurological symptoms. In cases where symptoms were present on discharge, follow-up visits were analyzed to approximate the estimated total duration.

Within our cohort of patients, two patients had previously been enrolled under an IRB-approved protocol (2043GCCC, IRB HP-00091736) to assess for CAR-T cells in the cerebrospinal fluid (CSF.) Samples were collected for immunomonitoring of patients following CAR-T treatment, and those results are included in our findings. The methodology of this assay included staining of peripheral blood (PB) mononuclear cells from CSF. This was performed using a panel of monoclonal antibodies and CAR-T detection reagents following the manufacturer’s instructions. Live cells were identified by 7-AAD dye exclusion (Miltenyi, cat. no. 130-111-568). Samples were acquired using a Miltenyi MACSQuant Analyzer 10.

## Results

A retrospective chart review was conducted on 374 patients with MM or NHL who received CAR-T therapy at the University of Maryland Medical Center between the years of 2015-2025. Seventeen patients were excluded due to receiving investigational CAR-T products. Of the 357 remaining patients, 17 patients were identified as having non-ICANS neurological complications (patient presenting symptosm summarised in Table 1 and case details summarized in Tables 2 and 3).

**Table 2.** MM atypical CAR-T patients characteristics

Patient	CAR-T Product	Primary malignancy	CNS involvement or known CNS disease	CRS	Non-ICANS day	Non-ICANS duration	Treatment	Resolved on Discharge	Follow up studies	Non-ICANS Description	Comments
66 Female (D)	Ciltacabtagene autoleucel (Carvykti)	Multiple Myeloma	No CNS disease or CNS involvement of malignancy	No	16	11	Initial PO dexamethasone, IV solumedrol 5 days, 7 weeks later PO dexamethasone and IV solumedrol	No Estimated duration 50 days	MRI: Normal Basic LP counts: CART cells	Bilateral cranial nerve VII palsy	CART in CSF, GBS ruled out
59 Male (A)	Ciltacabtagene autoleucel (Carvykti)	Multiple Myeloma	No CNS disease or CNS involvement of malignancy	Yes	18	11	Dexamethasone PO, IV, and IT, Anakinra	Not present at discharge	MRI Brain unremarkable, Lumbar Puncture with CSF CAR-T Assay	Facial Diplegia originally, resolved	CART in CSF
42 Male (D)	Ciltacabtagene autoleucel (Carvykti)	Multiple Myeloma	No CNS disease or CNS involvement of malignancy	No	27	17	Oral Dexamethasone with prolonged taper	No Estimated duration 240 days	MRI: Normal	Unilateral CN VII palsy and bilateral UE tremors	Tremor resolved but facial palsy became mild but never completely resolved
81 Male (D)	Ciltacabtagene autoleucel (Carvykti)	Multiple Myeloma	No CNS disease or CNS involvement of malignancy	No	18	9	High dose steroids, limited by peripheral edema. Antipsychotics for delirium/agitation.	No Estimated duration 114 days, until patient’s death	MRI: Normal LP: Elevated protein and glucose	Bradykinesia, hypokinetic dysarthria, bilateral hand tremor, shuffling gait, and cogwheel rigidity all consistent with Parkinsonism’s which all persisted until the patient’s death	CART in CSF Time from CAR-T to death: 114 days
60 Female (A)	Ciltacabtagene autoleucel (Carvykti)	Multiple Myeloma	No CNS disease or CNS involvement of malignancy	No	21	18	IVIG, IV and PO dexamethasone, prolonged rehab admission	No Estimated duration 365 days	MRI: Spine MRI without TM, Brain nl LP: CART cells	Transverse Myelitis, presenting as LE > UE weakness, band-like sensation loss around trunk	CART in CSF TM Confirmed with EMG showing an axonal motor and sensory neuropathy consistent with transverse myelitis MRI can be negative in 70% TM cases

**Table 3.** NHL atypical CAR-T patients characteristics

Patient	CAR-T Product	Primary malignancy	CNS involvement or known CNS disease	CRS	Non-ICANS day	Non-ICANS duration	Treatment	Resolved on DC	Follow up studies	Non-ICANS Description	Comments
51 Female (A)	Axicabtagene ciloleucel (Yescarta)	DLBCL	No CNS disease or CNS involvement of malignancy	Yes	6	2	Dexamethasone	Yes	MRI: Negative, stroke workup negative. EEG: Mild slowing	Left CN VII palsy and left arm weakness	
60 Male (A)	Axicabtagene ciloleucel (Yescarta)	DLBCL	No CNS disease or CNS involvement of malignancy	No	6	7	Dexamethasone, Tocilizumab	Yes	MRI: Normal, paraneoplastic (including MG) panel negative)	Cranial nerve VI palsy	Resolved with dexamethasone
60 Male (D)	Axicabtagene ciloleucel (Yescarta)	DLBCL	Metastatic CNS disease with prior stroke, epilepsy, and SDH	Yes	1	18	IV Dexamethasone	No Estimated duration 150 days	MRI: Progressive periventricular leukoencephalopathy EEG: Moderate slowing	Expressive aphasia and persistent encephalopathy	
82 Male (A)	Axicabtagene ciloleucel (Yescarta)	DLBCL	No CNS disease or CNS involvement of malignancy	Yes	4	11	Dexamethasone, Methylprednisone	Yes	CT Scan x 3	Expressive aphasia with preserved comprehension and command following	Was distinct aphasia different from conventional ICANS delirium
71 Female (A)	Axicabtagene ciloleucel (Yescarta)	DLBCL	No CNS disease or CNS involvement of malignancy	No	5	4	Dexamethasone, Tocilizumab	Yes	MRI: Normal	Bradykinesia, increased speech latency, and slow stooped posture consistent with Parkinsonism’s, improved with steroids	
49 Male (D)	Lisocabtagene maraleucel (Breyanzi)	DLBCL	Metastatic CNS disease s/p WBR with metastatic brain lesions	No	4	46	IV dexamethasone followed by IV Solumedrol	No Estimated duration 54 days	MRI: New FLAIR hyperintensity with mass effect EEG: Moderate slowing	Progressive leptomeningeal T2/FLAIR hyperintensity with profound and protracted expressive aphasia. Discharged to hospice from CAR-T admission	Figure 1 Time from CAR-T to death: 54 days
57 Male (D)	Axicabtagene ciloleucel (Yescarta)	DLBCL	No CNS disease or CNS involvement of malignancy	Yes	5	52	IV Dexamethasone and Tocilizumab	No Estimated duration 90 days	MRI: Leukoenceph- alopathy EEG: Moderate slowing LP: HHV6 on PCR*	Grade IV toxicity requiring intubation, sedation, and paralysis with prolonged neurotoxicity. Discharged to hospice	Figure 2 *HHV-6 PCR positive in CSF; clinically consistent with latent reactivation rather than active encephalitis given lack of limbic involvement on MRI and low viral titers Time from CAR-T to death: 90 days
66 Female (D)	Axicabtagene ciloleucel (Yescarta)	DLBCL	Prior stroke. No CNS involvement of malignancy	Yes	3	52	Methylprednisolone, dexamethasone, tocilizumab, sultuximab, anakinra, rabbit ATG	No Estimated duration 85 days	MRI: Progressive bilateral T2 subcortical hyperintensity over the course of 10 days	Grade IV ICANS requiring intubation, sedation, progressed until the patient proceeded with hospice care	Progressive mental status changes, no improvement, patient died. Figure 3 Time from CAR-T to death: 85 days
75 Male (A)	Axicabtagene ciloleucel (Yescarta)	Follicular lymphoma	No CNS disease or CNS involvement of malignancy	Yes	2	1	IV Dexamethasone and Tocilizumab	Yes	None	Hallucinations of Aztec and Mayan masks without frank delirium or altered mental status. Patient cognizant of hallucinations	
73 Male (D)	Axicabtagene ciloleucel (Yescarta)	DLBCL	No CNS disease or CNS involvement of malignancy	Yes	3	7	IV Dexamethasone	No Estimated duration 4 years	MRI: Normal	Isolated Anterograde amnesia that persisted until the patient’s death 4 years after CAR-T	
62 Female (D)	Axicabtagene ciloleucel (Yescarta)	DLBCL	No CNS disease or CNS involvement of malignancy	Yes	1	17	IV Dexamethasone and Tocilizumab	No Estimated duration 30 days	MRI: New infarcts EEG: Moderate slowing	Non-occlusive cerebral venous thrombosis, separate ischemic stroke. Altered mental status, aphasia, and progressive severe functional decline leading to a transition to hospice care	Initially improved without intervention but then worsened and did not improve again Time from CAR-T to death: 30 days

A key differentiation of this series is that the 17 identified patients either: 1. had uncategorized neurologic symptoms while not concurrently meeting the American Society for Transplantation and Cellular Therapy (ASTCT) consensus criteria for classic ICANS or 2. Had additional imaging findings beyond the ASTCT definition of ICANS. This clinical divergence is the primary rationale for our characterization of these cases as distinct, non-ICANS neurological complications.

*Demographics:* The median age of identified patients with non-ICANS neurological complications was 64 years. Ten patients were male and 7 were female. Thirteen patients were white, 3 patients were African American, Hispanic, or Latino, and 1 was Asian.

*Malignancy characteristics:* A majority of patients had NHL as the primary malignancy (12 patients). Among them, 11 had diffuse large B-cell lymphoma (DLBCL) and 1 had follicular lymphoma. The remaining 5 patients had MM. Two patients had evidence of CNS metastatic disease prior to receiving CAR-T. One patient had a history of stroke and epilepsy prior to CAR-T. The remaining patients had no preexisting CNS disease. The most common CAR-T product was axicabtagene ciloleucel (11 patients), followed by ciltacabtagene autoleucel (5 patients) and lisocabtagene maraleucel (1 patient).

*Clinical course and outcomes:* During the initial hospitalization, 9 patients had concurrent or preceding CRS, all occurring in the NHL cohort. The median onset of the neurological complication was 4.5 days after CAR-T, with a median duration of 14 days. Only 5 patients had complete symptom resolution, while 11 patients had residual symptoms upon discharge. One patient developed symptoms after discharge. For patients who experienced clinical improvement, the overall time to symptom resolution ranged from 1 to 18 days (median, 11 days), varying by complication type (individual recovery tracking detailed in Table 1).

**Table 1.** Presenting symptoms of non-ICANS

Syndrome	*N* (%)	Representative features	Imaging/CSF findings	Treatment	Outcome
Cranial neuropathies	4 (23.5%)	2 facial diplegia, 1 unilateral facial palsy, 1 abducens palsy	Normal brain MRI; CSF negative for carcinomatosis but + CAR-T cells	Corticosteroids (PO/IV); 1 received IT steroids + anakinra	Full/partial recovery; 2 protracted
Aphasia	2 (11.8%)	Expressive aphasia with preserved comprehension	MRI: global volume loss (1); CTs unrevealing (1)	Corticosteroids	Resolution over protracted course
Movement disorders (MNT)	2 (11.8%)	Bradykinesia, tremor, shuffling gait, rigidity; stooped posture, speech latency	Normal MRI; 1 CSF normal	Corticosteroids	Resolution in 4-9 days
Leukoencephalopathy	4 (23.5%)	Progressive cognitive decline, white matter disease	Serial MRI: diffuse/periventricular/brainstem/subcortical T2 hyperintensities	Corticosteroids, tocilizumab, siltuximab, anakinra, ±ATG	3 poor outcomes
Psychiatric syndromes	2 (11.8%)	Visual hallucinations with preserved orientation (1); persistent anterograde amnesia (1)	MRI/CT unrevealing	Corticosteroids	Hallucinations resolved; amnesia persisted until death
Vascular syndromes	2 (11.8%)	1 venous sinus thrombosis + arterial stroke; 1 TIA-like syndrome	MRI/CT: stroke and venous thrombosis (1); negative imaging (1)	Corticosteroids	Stroke case: poor outcome; TIA-like: resolved
Other (transverse myelitis)	1 (5.9%)	Bilateral limb weakness, sensory level T6-T10	MRI negative; CSF with CAR-T cells; NCS: axonal motor-sensory neuropathy	Corticosteroids + IVIG	Partial improvement; protracted recovery

Neurological manifestations varied: 5 patients experienced myelopathy or neuropathy as their primary symptom, 2 had Parkinsonism, and 3 patients developed neuropsychiatric symptoms, including hallucinations, amnesia, depression, and labile mood. Ten patients had a prolonged duration of symptoms, defined as lasting more than 30 days. Five patients developed severe refractory encephalopathy; all of them transitioned to hospice care and subsequently died.

**Figure 1.**
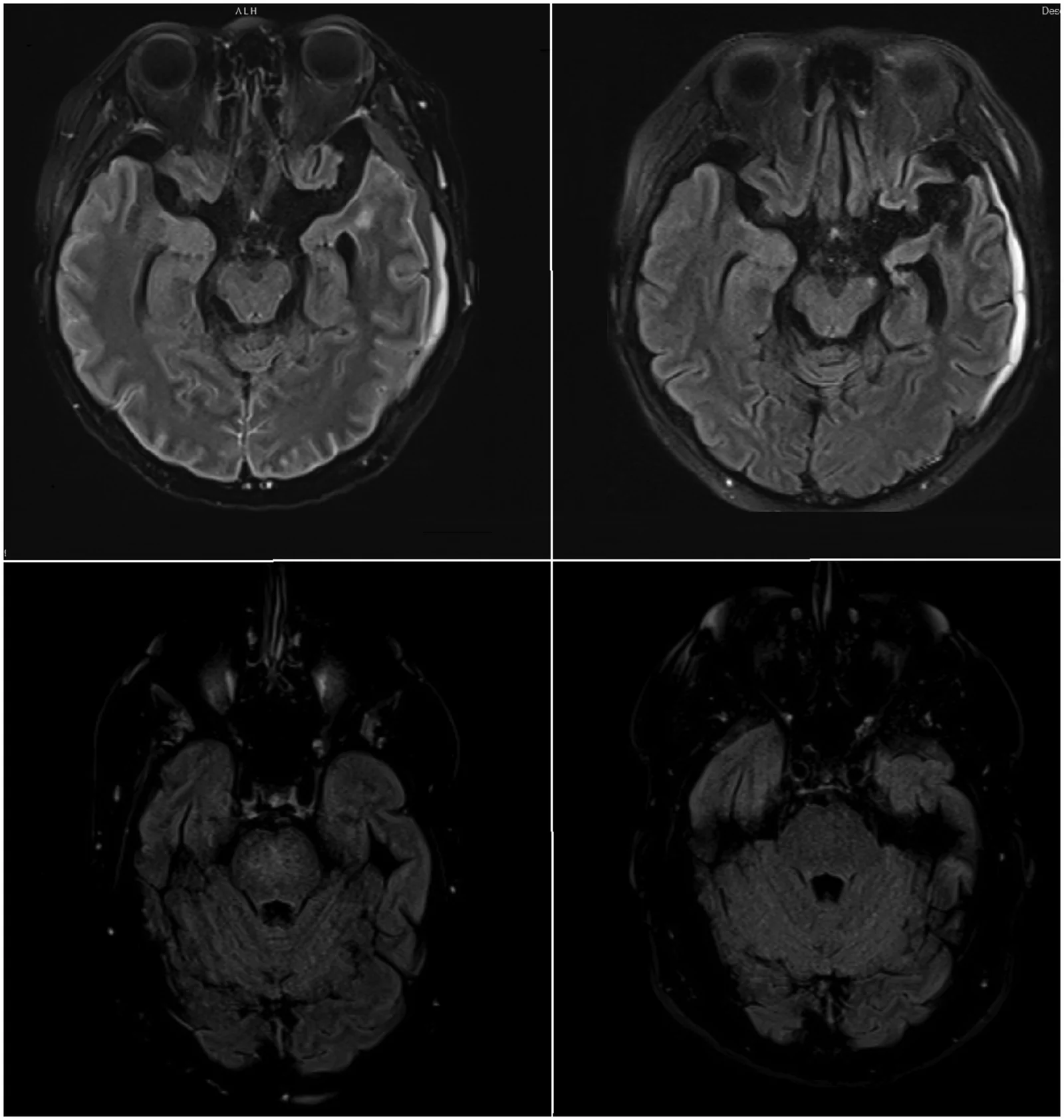
Top: MRI T2/FLAIR sequence showing the diffuse T2 leptomeningeal hyperintensity (left image 30 days post-CAR-T and right pre-CAR-T). Bottom: MRI T2/FLAIR sequence showing the progressive T2 brainstem hyperintensity (left post-CAR-T, right pre-CAR-T).

**Figure 2.**
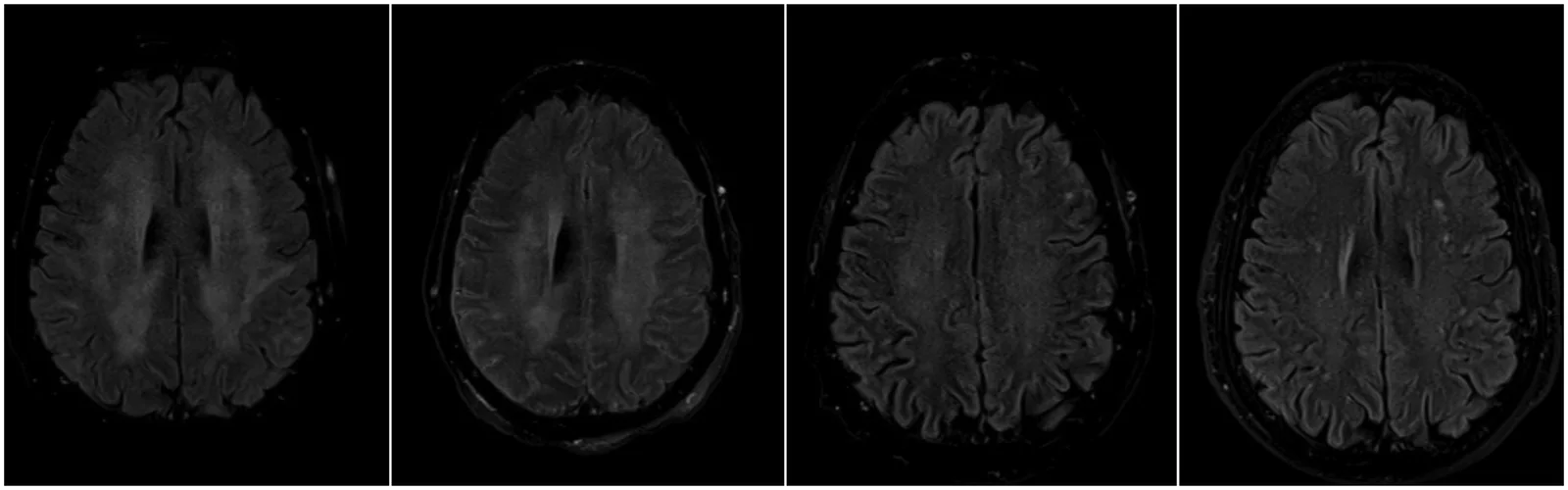
MRI T2/FLAIR sequence showing the progressive subcortical white matter leukoencephalopathy (leftmost image is 10 days post-CAR-T, right most image is pre-CAR-T).

Fifteen patients received a follow-up brain MRI, of which 5 demonstrated abnormal findings (Figures 1 and 2), including new FLAIR hyperintensity with mass effect (*n* = 1), new cerebral infarcts (*n* = 1), and leukoencephalopathy/encephalomalacia (*n* = 3). No patients developed EEG evidence of seizure activity, although 5 patients developed mild-to-moderate slowing on electroencephalogram (EEG).

**Figure 3.**
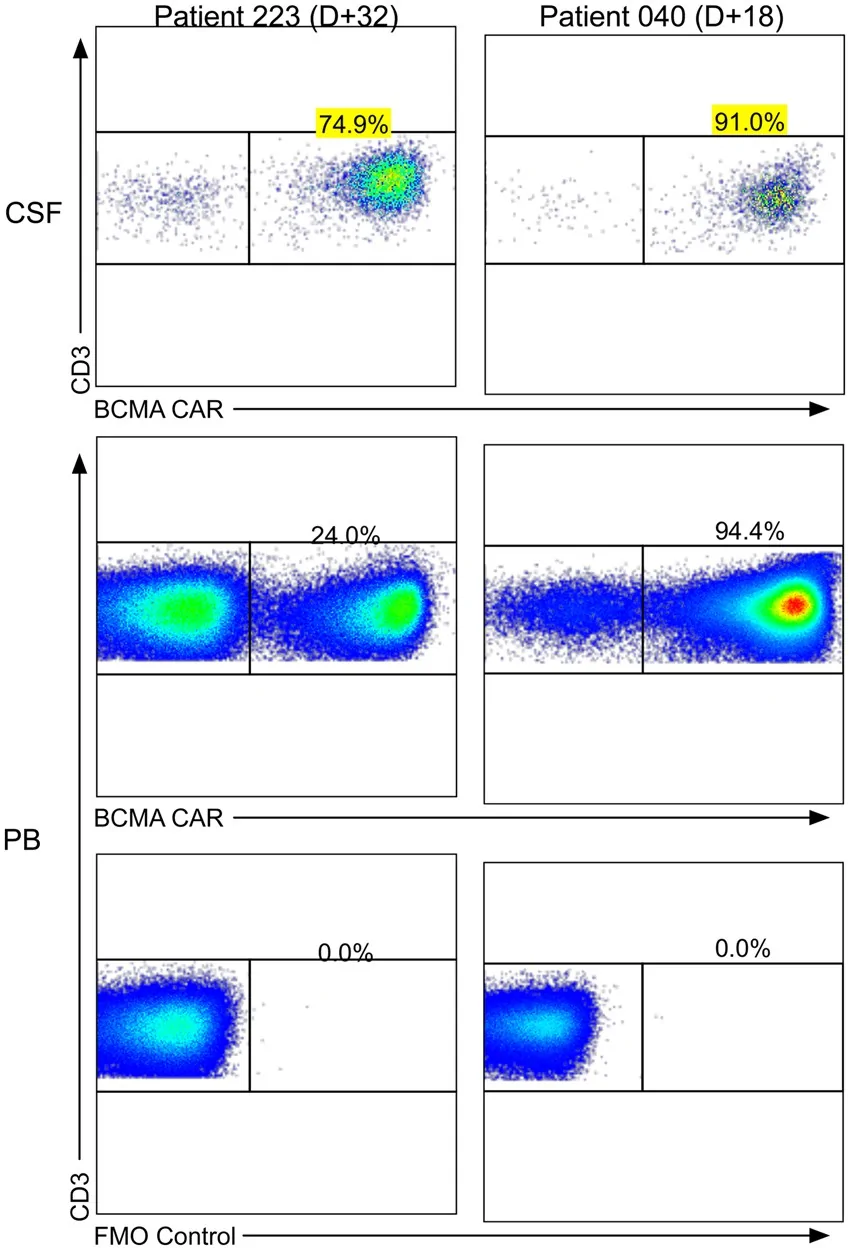
Enrichment of CAR T cells in the patients’ CSF. Analysis of BCMA-targeted CAR T cell numbers in the PB and the CSF after CART infusion. Analyses were performed by flow cytometry following staining with BCMA CAR detection reagent (Miltenyi, Cat# 130-133-888), which represents a fluorescent, full-length, recombinant BCMA protein binding to the CAR expressed on the cell surface, and co-staining with anti-CD3 and other T cell markers. Dot plots (two upper rows) show percentages of CAR-expressing T cells vs. all T cells in the patient’s PB (upper row) and CSF (middle row), respectively, at different timepoints. To control for unspecific background staining a Fluorescence Minus One (FMO) control was used (lower row).

**Figure 4.**
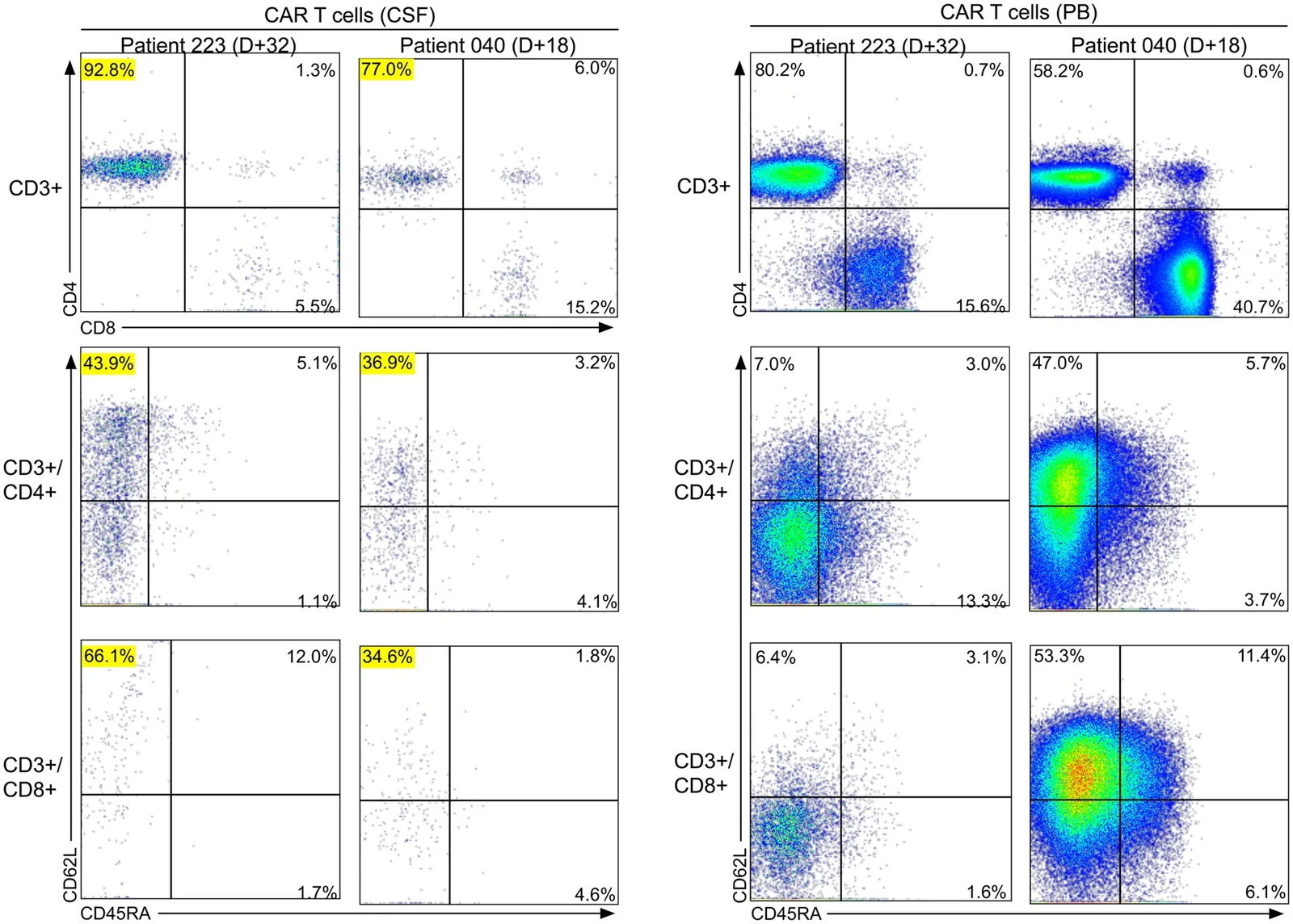
CAR T cell treatment leads to an enrichment of CD4^+^ central memory-type of CART in the CSF of patients without CNS disease. CAR T cell CD4^+^ and CD8^+^ subtypes were quantified at the different timepoints, and CAR T cell memory subtypes were determined on CD4^+^ and CD8^+^ CAR T cells, respectively, by co-staining for CD45RA and CD62L in both the CSF (left) and the PB (right).

*CSF and serum CAR-T analysis:* Two patients with severe encephalopathy underwent lumbar puncture (LP) for CSF evaluation. Both were MM patients. CSF was collected on day 32 for the first patient and day 18 for the second. Using the in-house assay, numbers of circulating CAR T-cells were determined by quantifying frequencies of T cells expressing the BCMA CAR in the PB vs. the CSF. In both cases, CAR-T levels were significantly higher in the CSF compared to the same patient’s PB from the same day (Figure 3). Furthermore, we observed an enrichment of CD4+ CAR T cells in the CSF vs. the PB, and CAR T cells in the CSF, especially in patient 223, were more likely to express a central memory (CM; CD62L + CD45RA-) phenotype (Figures 3 and 4).

In one patient, HHV-6 DNA was detected in the CSF. The lack of classic limbic involvement on MRI and low viral titers led the clinical team to favor latent reactivation rather than active encephalitis. We acknowledge that under the 2017 *European Conference on Infections in Leukemia* (ECIL-7) guidelines, up to 60% of true HHV-6 encephalitis cases can lack classic limbic findings. Therefore, active HHV-6 encephalitis remains a possible alternative etiology or confounding factor in this patient’s clinical presentation.

*Treatment:* Seven patients required intensive care unit (ICU) level of care for management. Corticosteroids (dexamethasone or methylprednisolone) and tocilizumab were the most frequently administered therapies, with 14 patients receiving both. When converted to prednisone equivalents, the median cumulative steroid dose was 3,164.5 mg. Additional immunomodulatory therapy included siltuximab (4 patients), anakinra (3 patients), intra-thecal steroids (1 patient), and intra-thecal chemotherapy (1 patient).

*Comparative cohort context:* To contextualize the 17 patients identified with non-ICANS neurological complications, we compared their profile to the broader cohort of 357 patients treated with commercial CAR-T products at our center. The baseline demographics and toxicity rates of our total 357-patient population align with established benchmarks for large academic cell therapy centers. We additionally compared patients with non-ICANS (*n* = 17) to those with conventional ICANS (*n* = 120) and patients without ICANS (*n* = 220) (Table 4). Compared with patients without ICANS, non-ICANS cases demonstrated a higher incidence of CRS (88.2% vs 69.1%) and more frequent ICU transfer (35.3% vs 7.7%). Compared with conventional ICANS, non-ICANS occurred in a cohort with a greater proportion of multiple myeloma patients (35.3% vs 19.2%) and a higher representation of ciltacabtagene recipients (23.5% vs 4.2%). CRS incidence was similar between non-ICANS and conventional ICANS cohorts; however, grade ≥3 CRS was uncommon among non-ICANS cases. ICU transfer occurred less frequently compared with conventional ICANS (35.3% vs 54.2%).

**Table 4.** Comparison between non-ICANS cohort, ICANS cohort, and no ICANS cohort

Characteristic	No ICANS (*n* = 220)	Conventional ICANS (*n* = 120)	Non-ICANS (*n* = 17)
Multiple myeloma, *n* (%)	63 (28.6)	23 (19.2)	6 (35.3)
Lymphoma, *n* (%)	157 (71.4)	97 (80.8)	11 (64.7)
Axi-cel, *n* (%)	107 (48.6)	75 (62.5)	10 (58.8)
Tisa-cel, *n* (%)	4 (1.8)	2 (1.7)	0
Brexu-cel, *n* (%)	14 (6.4)	10 (8.3)	0
Cilta-cel, *n* (%)	21 (9.5)	5 (4.2)	4 (23.5)
Any CRS, *n* (%)	152 (69.1)	110 (91.7)	15 (88.2)
Grade ≥3 CRS, *n* (%)	4 (1.8)	14 (11.7)	0
ICU transfer, *n* (%)	17 (7.7)	65 (54.2)	6 (35.3)
Death during follow-up*, *n* (%)	3 (1.4)	10 (8.3)	0

*Footnote: Given the small number of Non-ICANS cases, formal statistical testing was not performed. Comparisons are presented descriptively and should be considered hypothesis-generating rather than confirmatory*.

*Demographics:* The median age of the full cohort was 62 years, with a distribution of 72% White and 18% African American patients, mirrors the 17-case subset (median age 64; 76.5% White).

*Product distribution:* Approximately 74% of our total patients received CD19-directed therapy for NHL, and 26% received BCMA-directed therapy for MM, consistent with the proportions seen in our non-ICANS subgroup (70.6% NHL; 29.4% MM).

*Standard toxicity:* Literature reports for similar cohorts indicate a CRS incidence of 70%-80% and a classic ICANS incidence of 30%-40%.^7^^, ^^8^ In contrast, our identified non-ICANS complications occurred in 4.76% of the total population. While the timing of symptom onset for our 17 cases (median 4.5 days) was similar to the 5-7 day window typically reported for classic ICANS, the clinical course differed significantly. Classic ICANS typically resolves within 7-9 days. However, our non-ICANS cohort demonstrated a markedly prolonged median duration of 14 hospital days, with 64.7% of patients experiencing residual deficits at the time of discharge.

We categorized the non-ICANS neurological complications into several distinct phenotypes (Table 1).

### Cranial Neuropathies

A total of 4 patients presented with cranial nerve palsies: 2 with facial diplegia, another with unilateral facial nerve palsy, and one with abducens nerve palsy. All patients had normal MRIs of the brain. Two patients had CSF analysis with flow cytometry, which ruled out leptomeningeal carcinomatosis but did reveal circulating CAR-T cells. The patient with the abducens nerve palsy had a myasthenia gravis workup, including paraneoplastic evaluation, including anti-acetylcholine receptor antibodies and muscle-specific tyrosine kinase. All patients had full or partial recovery with corticosteroids, albeit two with protracted recovery. One patient additionally received intrathecal corticosteroids and systemic anakinra. These cases illustrate how isolated cranial neuropathies can mimic CNS carcinomatosis or neuromuscular disorders such as myasthenia gravis.

### Aphasia

Two patients with NHL developed expressive aphasia. Unlike the generalized language deficits or confusion sometimes seen during typical ICANS-related delirium, both of our patients had preserved comprehension and were able to follow complex commands but showed markedly impaired spontaneous speech. This was determined by expert neurologic evaluation from the in-hospital neurology consult service, but no formal language assessment or neuropsychological testing was performed. All patients with aphasia had EEG monitoring to rule out NCSE. Both patients had diffuse slowing, but none had findings of NCSE. One patient had a brain MRI showing global volume loss without ischemia. The other had repeated head CT scans (3 in total), all unrevealing. Both improved with corticosteroids, though over a protracted course. Isolated aphasia is concerned for an acute dominant frontal lobe cortical stroke, though neither patient had hemiparesis. Both patients underwent appropriate stroke rule-out brain imaging, including neurovascular imaging.

### Movement Disorders

Two patients developed Parkinsonism, one MM patient and one NHL patient. Drug-induced Parkinsonism was ruled out, as none of the patients were being treated with medications typically associated with this syndrome. The MM patient presented with typical Parkinsonism features with bradykinesia, tremor, shuffling gait, and cogwheeling rigidity. The NHL patient had bradykinesia, stooped posture, and increased speech latency. Neither patient had documented micrographia. Both patients had normal brain MRIs, and one patient had a normal CSF evaluation, including cell counts, protein, glucose, and flow cytometry. Both improved relatively quickly with corticosteroids, in 9 and 4 days, respectively. Parkinsonism is increasingly recognized as a potential CAR-T related complication, particularly with BCMA-targeted therapies.^16^ BCMA is expressed on neurons and astrocytes in the basal ganglia^17^; however, while some reports note irreversible BCMA-related Parkinsonism, the rapid reversibility in our cohort may suggest a potentially distinct underlying mechanism or the benefit of early immunosuppressive intervention.

### Leukoencephalopathy

Four NHL patients developed progressive leukoencephalopathy on serial brain MRIs compared with pre-CAR-T imaging. The MRI findings included diffuse T2 hyperintensities localizing to the subcortical, periventricular, and brainstem white matter (Figures 1 and 2). Three of the 4 patients had poor outcomes. All patients had extensive workups to rule out other causes. These patients received the most aggressive immunosuppressive therapies in our cohort. In addition to corticosteroids and tocilizumab, they were collectively treated with siltuximab, anakinra, and one patient with rabbit-derived thymocyte immunoglobulin (ATG rabbit). These findings suggest CD19-targeting CAR-T therapies may provoke diffuse autoimmune injury to CNS white matter tracts.

### Psychiatric

Two patients developed distinct psychiatric symptoms separate from the characteristic delirium seen in ICANS. One patient experienced fully formed visual hallucinations while being fully oriented and cognizant that the visuals were in fact hallucinations. Symptoms resolved rapidly with corticosteroids. The second patient developed anterograde amnesia without other cognitive deficits, which lasted until the patient’s death 4 years later. Differentiating such cases from in-hospital delirium, sundowning, and toxic-metabolic encephalopathy remains challenging in an aging population with significant comorbidities.

### Vascular

Two patients had vascular neurologic syndromes. The first developed non-occlusive cerebral venous sinus thrombosis and a separate arterial ischemic stroke. These vascular findings were concurrent with symptoms consistent with typical ICANS. However, the ICANS symptoms were not consistent with the location and small size of the ischemic stroke, so it was believed to be separate etiologies. The second developed left face and arm weakness acutely, though neuroimaging was negative and symptoms resolved with corticosteroids. A clear etiology was not identified, but transient ischemic attack was believed to be the etiology. A full stroke workup was negative. Vasculitis remains a possible etiology given the response to corticosteroids.

### Other Syndromes

One MM patient treated with BCMA CAR-T developed clinical transverse myelitis. She presented with bilateral arm and leg weakness, more pronounced in the legs. She also had a band-like loss of sensation in the T6-T10 dermatome. She had an unrevealing full CNS MRI. CSF flow cytometry and cytology analyses were performed and were notable for the presence of CAR-T cells. A nerve conduction study showed an associated axonal motor and sensory peripheral neuropathy, complicating the clinical picture of her suspected central nervous system disorder. The patient was treated with corticosteroids and intravenous immunoglobulin, had mild improvement, and was discharged to rehab. There was a protracted recovery over the following year. Though the imaging was negative, it is reported that MRI imaging can be negative in up to 40% of transverse myelitis cases.^18^

### Deaths

Among the 5 patients who developed severe refractory encephalopathy and subsequently died, the time from CAR T-cell administration to death ranged from 30 to 114 days (median, 85 days).

## Discussion

This retrospective review highlights non-ICANS neurological complications at a high-volume academic cell therapy center. Classic features of ICANS, such as tremors, delirium, and global encephalopathy, are well characterized and incorporated into treatment algorithms. Our findings demonstrate that distinct, non-classic neurotoxicities also occur. As CAR-T therapy continues to expand, familiarity with these diverse manifestations will be increasingly important. Early identification could reduce delays in immunosuppressive treatment and avoid unnecessary, often costly, diagnostic workups.

In the current cohort, non-ICANS neurological complications occurred in 17 patients. While the majority of these patients had NHL, this reflects the overall proportion of patients treated at our center during this timeframe. Similarly, no single CAR-T product appeared to predispose patients to these presentations.

Concurrent CRS and preexisting neurologic disease are known risk factors for typical ICANS.^19^ In our series, over half the patients had concurrent or preceding CRS. Only 2 of our patients had a prior history of CNS metastases, and only one patient had prior non-malignancy-related CNS disease. While our small sample size limits definitive conclusions, these findings suggest that neither CRS nor CNS involvement is uniquely predictive of these non-classic neurotoxicities.

The time to onset (median 4.5 days after CAR-T) was not a significant deviation from the 5-7-day window of typical ICANS.^20^ However, the duration of symptoms was notably prolonged, with a median duration of 14 hospital admission days and total symptom durations greater than 30 days for a majority of patients. It is doubtful that a delay in diagnosis and treatment alone fully explains this protracted duration, raising the possibility of a distinct pathophysiologic mechanism, such as persistent localized immune activation or cellular infiltration within the CNS sanctuary.

Our observed incidence of 4.76% for non-ICANS neurological complications aligns closely with emerging multicenter data and registry reports characterizing these distinct phenotypes. Recent studies have reported rates of MNT toxicities ranging from 1% to 5% in patients receiving BCMA-targeted therapies.^14^^,^^16^ Similarly, the incidence of isolated cranial neuropathies or peripheral neuropathy reported as 6% across large NHL cohorts.^12^ Though, it is noted that other non-ICANS toxicities such as TIAN have been reported as high as 20% in patients with CNS lymphoma^21^ and this underscores the need for larger prospective studies to delineate risk factors.

While our single-center rate is consistent with these figures, the high frequency of prolonged symptoms (58.8%) and residual deficits (64.7%) in our cohort suggests that while these events are relatively rare, their clinical impact is disproportionately severe compared to classic, reversible ICANS. This emphasizes that non-ICANS complications are not merely “atypical variants” of a common syndrome but represent a clinically significant subset of neurotoxicity requiring specific diagnostic vigilance.

Patients with focal neurologic deficits (such as isolated cranial neuropathies, myelopathy, and aphasia) often presented with normal neuroimaging and CSF studies. This necessitated extensive workups to exclude alternative etiologies, including infection, leptomeningeal disease, stroke, or paraneoplastic syndromes. A critical limitation in evaluating these syndromes is the difficulty of distinguishing true causal inference from mere temporal association with CAR-T administration, particularly given the extensive prior therapies and complex baseline medical statuses of these patients.

Despite these confounding factors, the presence of these focal findings may suggest targeted neuroinflammation moving beyond the traditional model of global blood-brain barrier disruption. In particular, the development of progressive leukoencephalopathy in patients receiving CD19-targeted therapy supports the hypothesis of diffuse immune-mediated injury affecting white matter tracts. At our institution, our baseline brain MRI data allowed us to identify new leukoencephalopathy relative to the patient’s baseline, which is notable as many cell therapy centers do not utilize baseline brain MRIs.

Further research into the pathophysiology of these varied presentations is urgently needed. While BCMA receptors are expressed in specific areas of the brain,^14^^,^^17^ these non-ICANS complications were not restricted to BCMA-targeted CAR-T products. Furthermore, our flow cytometry data demonstrates an enrichment of CAR-T cells (specifically a central memory phenotype) in the CSF compared to the PB of 2 patients with severe encephalopathy. While these limited CSF findings do not establish regional trafficking or structural targeting on their own, they generate the hypothesis that certain neurotoxicities may involve the active trafficking of immune cells into the CNS compartment.

### Limitations

Our study has several limitations. The small sample size of 17 patients limits statistical power and precludes definitive correlation with specific CAR-T products, patient demographics, or preexisting conditions. Our findings are descriptive and hypothesis-generating, rather than inferential. As a retrospective chart review spanning a 10-year timeframe, the study is subject to temporal heterogeneity, which may influence the detection, grading, and classification of neurological complications as institutional practices and standardized definitions have evolved. Furthermore, the reliance on heterogeneous diagnostic workups performed by multiple clinical teams means that additional etiologies (eg, subclinical seizures, medication-induced delirium) may have been under-recognized. Finally, categorizing these heterogeneous cases inherently risks oversimplification, underscoring the need for prospective, multi-center registries utilizing standardized diagnostic protocols to validate our observations.

## Conclusions

CAR-T therapy remains a transformative modality, but its success has illuminated a broader spectrum of neurotoxicities than originally described. This single-center retrospective review identified a cohort of 17 patients who developed non-ICANS neurological complications, including focal neurological deficits and distinct neurocognitive syndromes. These manifestations present significant diagnostic challenges, often requiring extensive workups to rule out infection, stroke, or disease relapse. Increased clinical vigilance for these diverse syndromes is essential for prompt intervention with immunosuppressive therapy, which may mitigate severe and persistent morbidity. Future multi-institutional studies are warranted to investigate the distinct underlying pathophysiology of these subgroups and to develop prognostic biomarkers for this severe subset of CAR-T neurotoxicity.

## Data Availability

The data that support the findings of this study are available from the corresponding author upon reasonable request.
